# Supplementary material for: Active droploids
Source: Nat Commun. 2021 Oct 14;12:6005. doi: 10.1038/s41467-021-26319-3 (PMC8516867; doi:10.1038/s41467-021-26319-3)
Supplement: Supplementary file 1 — Supplementary information. [file 41467_2021_26319_MOESM1_ESM.pdf]

Supplementary Information
Active droploids

Jens Grauer,[1,*] Falko Schmidt,[2,*] Jesus Pineda,[2] Benjamin Midtvedt,[2] Hartmut Löwen,[1] Giovanni Volpe,[2] and Benno Liebchen[3,†]

[1]*Institut für Theoretische Physik II: Weiche Materie, Heinrich-Heine-Universität Düsseldorf, D-40225 Düsseldorf, Germany*
[2]*Department of Physics, University of Gothenburg, SE-41296 Gothenburg, Sweden*
[3]*Institut für Physik kondensierter Materie, Technische Universität Darmstadt, 64289 Darmstadt, Germany*

Here we provide supplementary information on experimental aspects such as the phase diagram of a water–2,6-lutidine mixture, the experimental setup, the influence of wetting properties on particle behaviour, and the segmentation and tracking of the droploids.

* These authors contributed equally.
† benno.liebchen@pkm.tu-darmstadt.de

SUPPLEMENTARY FIGURES

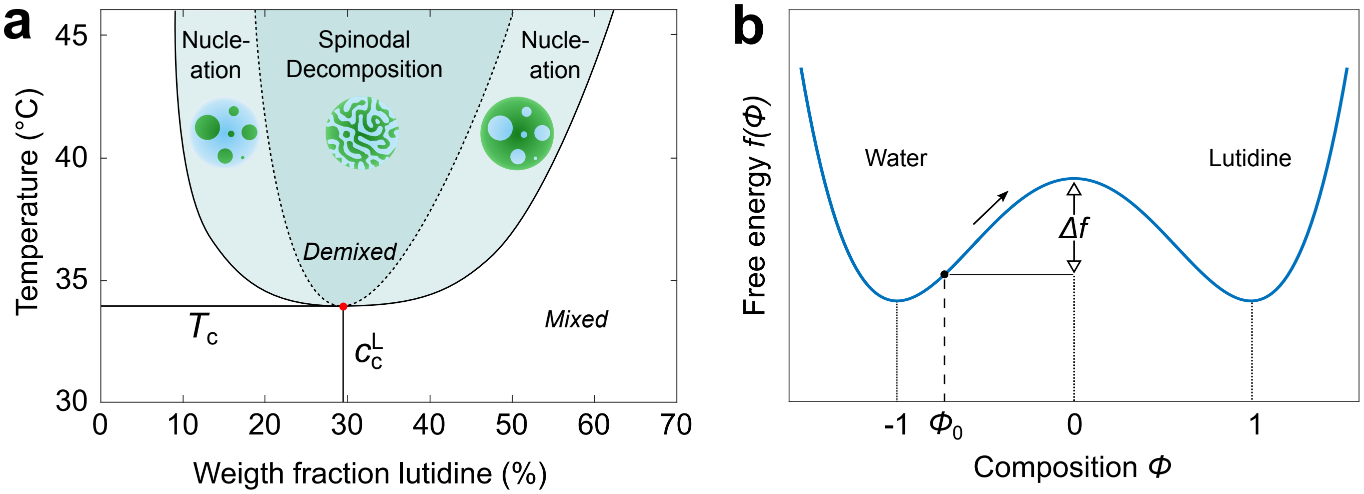

Supplementary Figure 1. **Phase diagram of the water–2,6-lutidine mixture and characteristic free energy.** **a** The water–2,6-lutidine mixture features a lower critical point at $T_c = 34.1°$ and $c_c^L = 28.4\%$, where the binodal (solid line) and the spinodal (dotted line) coincide. Upon heating, two phases with different concentrations occur, which are separated by a pronounced interface (blue: water-rich phase; green: lutidine-rich phase). Between the binodal and the spinodal droplets of the lower concentrated phase grow in the higher concentrated phase, continuously throughout the sample. Above the spinodal line, spinodal decomposition occurs, where both phases form symmetrical structures. **b** Schematic illustration of the free energy profile $f(\phi) = \frac{a}{2}(T - T_c)\phi^2 + \frac{b}{4}\phi^4$ of a binary mixture as a function of the composition ϕ. A free energy barrier of height Δf located at $\phi = 0$ must be crossed during a transition from ϕ_0 (water-rich region) to $\phi > 0$ (lutidine-rich region).

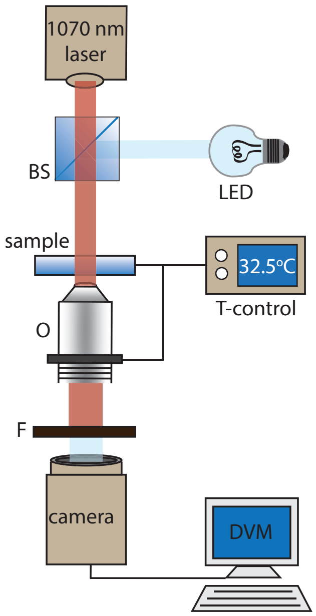

Supplementary Figure 2. **Schematic of the experimental setup.** The setup is a home-made version of an inverted microscope setup. The sample is confined in a quasi-2D space between a coverslip and a microscopic slide and separated by spacer particles ($R = 0.85 \pm 0.02\,\mu$m microParticles GmbH). A defocused laser ($\lambda = 1070\,$nm) heats up light-absorbing colloids and causes local demixing of the near-critical mixture. The sample's temperature is fixed close to the critical temperature at $T_0 = 32.5°$C using a two-stage controller system consisting of a copper-plate heat exchanger with a water bath (T100, Grant Instruments) and of two Peltier elements attached to the objective (O) in feedback with a temperature controller (TED45, Thorlabs). A background light source (LED) is coupled to the laser beam path using a 50:50 beamsplitter (BS) and illuminates the whole sample area. The scattered light is collected with a 100x oil-immersion objective (O, NA=1.30) and imaged onto a camera where the laser is blocked by a filter (F). Using digital video microscopy (DVM) the particle's motion is being tracked and analyzed.

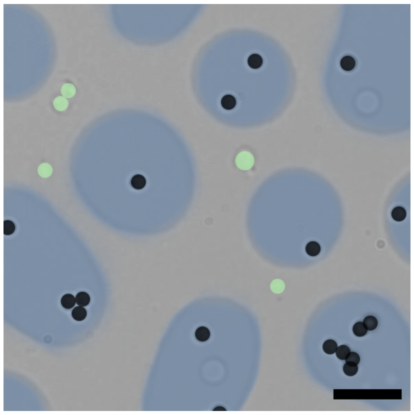

Supplementary Figure 3. **Influence of wetting properties on particle behaviour.** Whereas hydrophilic absorbing particles (black) are immersed inside water-rich droplets, hydrophobic non-absorbing particles (green) remain outside these regions. Scale bar represents $5\,\mu$m.

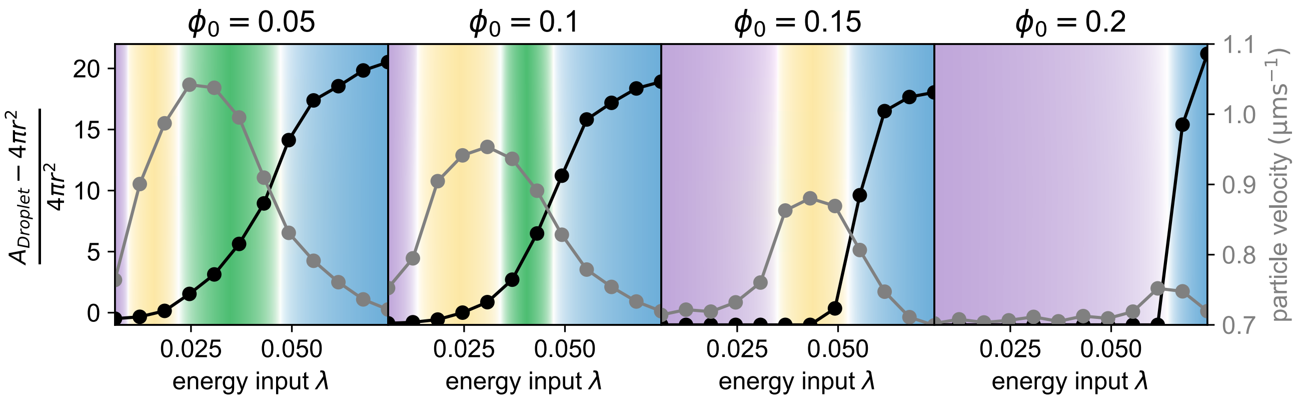

Supplementary Figure 4. **Quantitative phase characterization.** To distinguish between the different phases and the colors in Fig.2a we calculate the area with higher water concentration around the particles (where $\phi < 0$) and use a threshold criterion $A_{\text{Droplet}} > 8\pi r^2$ (or $(A_{\text{Droplet}} - 4\pi r^2)/(4\pi r^2) > 1$) to distinguish phases II and III (active and immotile droploids) from the other ones. This criterion requires that the water-rich area is larger than the area covered by a typical number of 8 colloids within a droplet at the time instance where the snapshots are evaluated. To distinguish phases II and III we calculate the mean particle velocity which indicates whether the appearing structures are active or passive. When the mean velocity clearly exceeds that of passive Brownian particles ($v > 0.8\,\mu\text{ms}^{-1}$, for the used sampling rate) we call the resulting structures active droploids (phase II). The data points shown here for different values of ϕ_0 and λ correspond to those from the phase diagram in Fig. 2a.

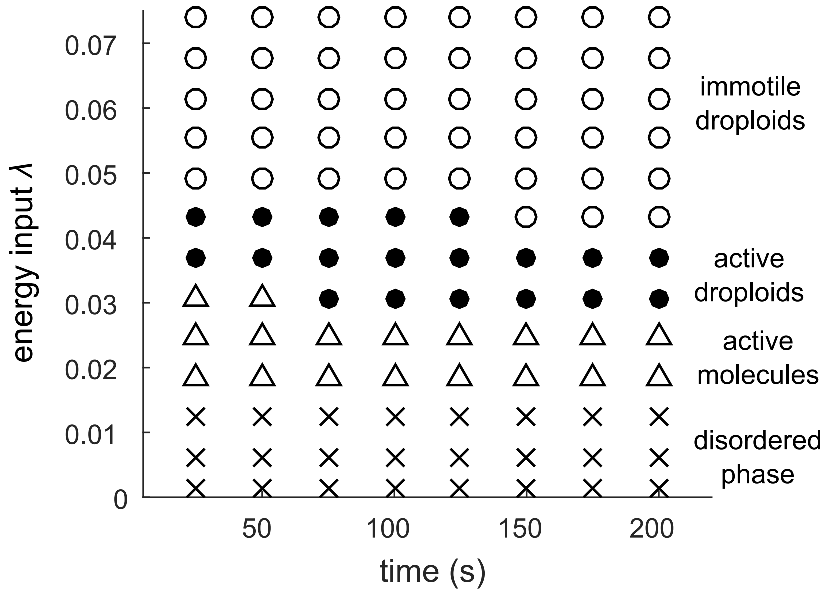

Supplementary Figure 5. **Time-evolution of phase boundaries:** Phases as a function of the net energy input λ and the time for a fixed averaged relative concentration difference from the critical point $\phi_0 = 0.1$. We find the same phases at all times and observe a slight evolution of the phase boundaries over time. The evaluated state points are indicated by crosses (disordered phase), triangles (active molecules), filled circles (active droploids), and empty circles (immotile droplets with particles at the interface). The quantitative criteria for the phases are given in Supplementary Fig. 4.

SUPPLEMENTARY METHODS

Droplet Segmentation

A deep-learning-based approach is used to detect the droplets and follow their morphology over time. Since droplets are never overlapping, the method is built around a binary classification of each pixel in an image into either background or droplet. The network architecture used to perform this task is similar to the U-Net [1], with a down-sampling step and a up-sampling step, and skip-connections there between (Supplementary Fig. 6a). The network was trained using simulated image-label pairs, generated by the deep learning framework DeepTrack 2.0 [2]. Examples are shown in Supplementary Fig. 6b-d.

Notably, the label was not constructed as a binary image directly, but as the distance transform of that binary

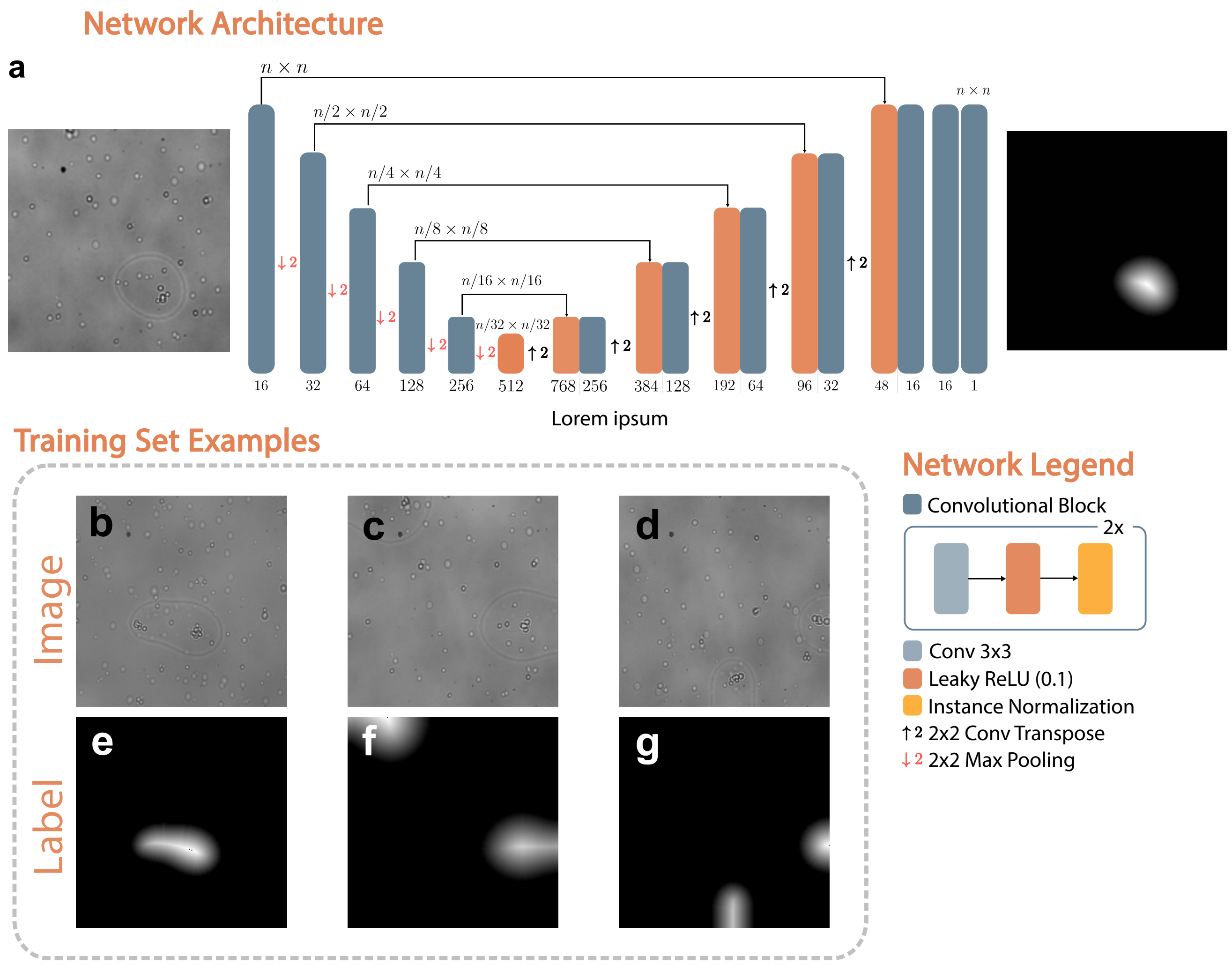

Supplementary Figure 6. **Overview of the deep-learning approach used to track and segment the droplets. a** The neural network architecture with an example input and output. The depth of the neural network was chosen as to give it a sufficient receptive field to analyze big droplets. Instance normalization is used to help propagate information deeper into the network. **b-d** Example simulated training images. The images are constructed by merging experimental backgrounds and droplets simulated by approximating the optical transfer function. **e-g** Training labels corresponding to the images in **b-d**. The labels are constructed by taking the distance transform of the binary image where the background is 0 and pixels inside a droplet are 1.

image, as can be seen in Supplementary Fig. 6e-g. In the experimental images, the inside of a droplet is essentially indistinguishable form the outside, and its classification can only be inferred from the surrounding droplet edges. As such, classifying the center of droplets becomes more difficult the larger the droplet is, because the information is further away in the image. We found that using the distance transform instead of the binary image helped the network learn to correctly detect the center of larger droplets, presumably because mistakes are punished more harshly in the training process.

Since the network is now trained on a regression problem, we use mean absolute error as loss function. Moreover, we used the Adam optimizer, with a learning rate of 0.0001. The network was trained for 100 epochs, each of which consisted of 1024 unique training samples split into batches of 8. Note that new training data was continuously generated during training.

A binary classification can be restored by thresholding the network output, from which individual droplets are detected using by finding connected regions of positive classification. Since the droplets are large and well separated, they are easily traced over time by their centroid.

Supplementary Figure 7 demonstrates the tracking of the network by showing input images next to the same image with the segmentation of each droplet overlaid, for a series of images taken from a experimental video. The images were chosen to demonstrate the correctness of the method in a few common scenarios, such as the merging of two droplets, the emergence of new droplets as well as densly populated images. The full tracked video is available as

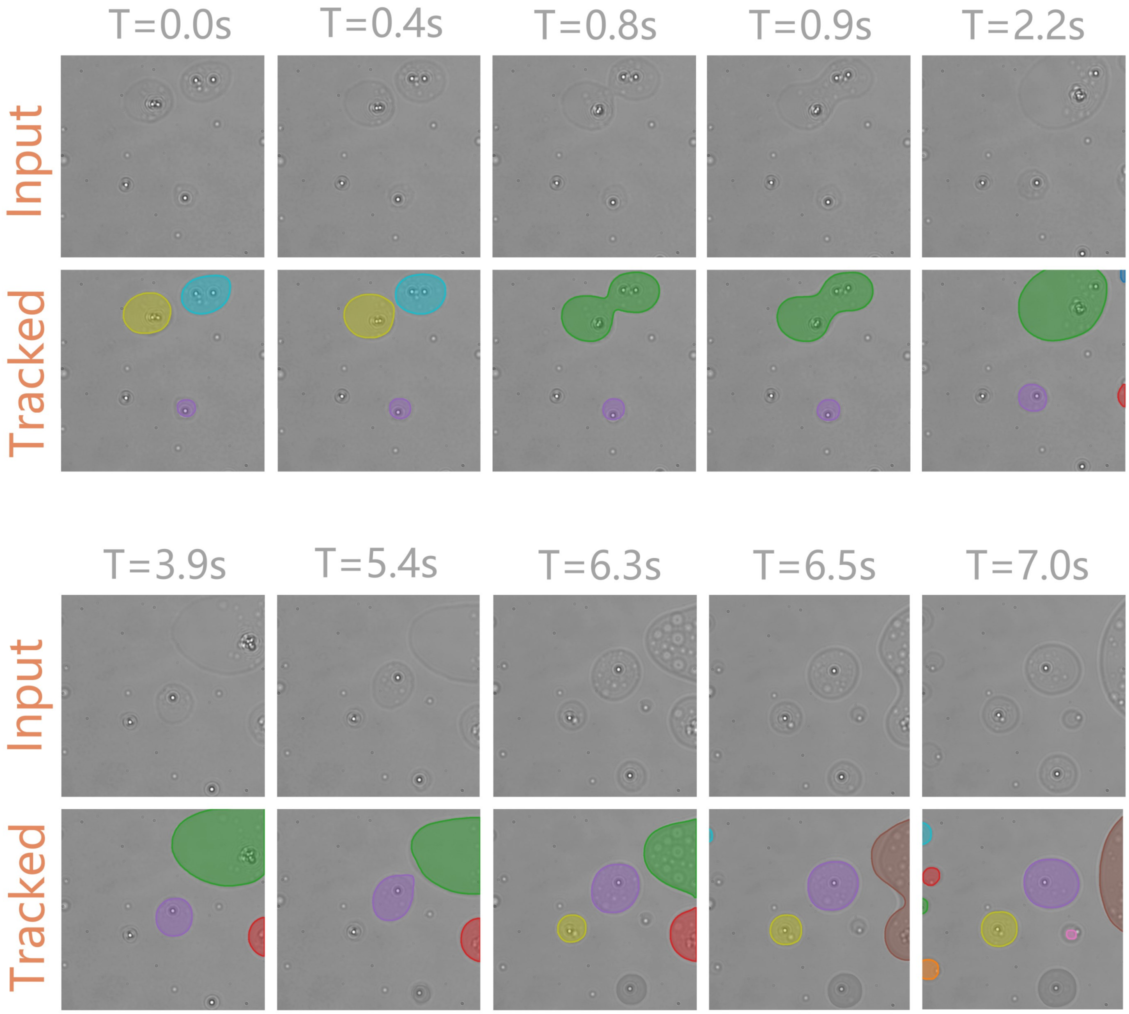

Supplementary Figure 7. **Example tracked droplets.** A few tracked frames from an experimental video, demonstrating the tracking using the deep learning approach. The top row shows raw images fed to to the tracking algorithm, while the bottom row shows the same images with the binary segmentation overlaid. The colors represent individual droplets and are consistent over time to show that the cells are correctly traced.

supplementary material (see Supplementary Movie 7).

SUPPLEMENTARY REFERENCES

[1] O. Ronneberger, P. Fischer, and T. Brox, U-net: Convolutional networks for biomedical image segmentation, in *Medical Image Computing and Computer-Assisted Intervention – MICCAI 2015*, edited by N. Navab, J. Hornegger, W. M. Wells, and A. F. Frangi (Springer International Publishing, Cham, 2015) pp. 234–241.

[2] B. Midtvedt, S. Helgadottir, A. Argun, J. Pineda, D. Midtvedt, and G. Volpe, Quantitative digital microscopy with deep learning, Appl. Phys. Rev. **8**, 011310 (2021).
