# Supplementary material for: Active droploids
Source: Nat Commun. 2021 Oct 14;12:6005. doi: 10.1038/s41467-021-26319-3 (PMC8516867; doi:10.1038/s41467-021-26319-3)
Supplement: Supplementary file 3 — Description of Additional Supplementary Files. [file 41467_2021_26319_MOESM3_ESM.pdf]

Description of Additional Supplementary Files

File Name: Supplementary Movie 1
Description: Experimental observations of the formation and movement of active droploids.

File Name: Supplementary Movie 2
Description: Simulations of the formation and movement of active droploids.

File Name: Supplementary Movie 3
Description: Explosive formation of a droploid in experiments.

File Name: Supplementary Movie 4
Description: Explosive formation of a droploid in simulations.

File Name: Supplementary Movie 5
Description: Formation of size-stabilized droplets with periodic light illumination in experiments.

File Name: Supplementary Movie 6
Description: Formation of size-stabilized droplets with periodic light illumination in simulations.

File Name: Supplementary Movie 7
Description: Segmentation and tracking of the droplets using a deep learning approach.
